# Investigating the Dissolution Performance of Amorphous Solid Dispersions Using Magnetic Resonance Imaging and Proton NMR

**DOI:** 10.3390/molecules200916404

**Published:** 2015-09-10

**Authors:** Francesco Tres, Steven R. Coombes, Andrew R. Phillips, Leslie P. Hughes, Stephen A. C. Wren, Jonathan W. Aylott, Jonathan C. Burley

**Affiliations:** 1 School of Pharmacy, Boots Science Building, University of Nottingham, Nottingham NG7 2RD, UK; E-Mail: Jon.Aylott@nottingham.ac.uk; 2 Pharmaceutical Development, AstraZeneca, Macclesfield SK10 2NA, UK; E-Mails: Steven.Coombes@astrazeneca.com (S.R.C.); andrew.r.phillips@astrazeneca.com (A.R.P.); Les.Hughes2@astrazeneca.com (L.P.H.); Stephen.Wren@astrazeneca.com (S.A.C.W.)

**Keywords:** bicalutamide, poorly soluble drugs, amorphous solid dispersions, hot melt extrusion, solid-state transformations, magnetic resonance imaging, suppressed water ^1^H-NMR

## Abstract

We have investigated the dissolution performance of amorphous solid dispersions of poorly water-soluble bicalutamide in a Kollidon VA64 polymeric matrix as a function of the drug loading (5% *vs.* 30% bicalutamide). A combined suite of state-of-the-art analytical techniques were employed to obtain a clear picture of the drug release, including an integrated magnetic resonance imaging UV-Vis flow cell system and ${}^{1}$H-NMR. Off-line ${}^{1}$H-NMR was used for the first time to simultaneously measure the dissolution profiles and rates of both the drug and the polymer from a solid dispersion. MRI and ${}^{1}$H-NMR data showed that the 5% drug loading compact erodes linearly, and that bicalutamide and Kollidon VA64 are released at approximately the same rate from the molecular dispersion. For the 30% extrudate, data indicated a slower water ingress into the compact which corresponds to a slower dissolution rate of both bicalutamide and Kollidon VA64.

## 1. Introduction

Due to an increasing number of poorly water-soluble molecules emerging from pharmaceutical pipelines, there is a continual drive to develop formulations for the delivery of such compounds [1,2]. Conventional formulations using the crystalline form of the drug often do not achieve the desired oral bioavailability and pharmacological effects. An alternative strategy is to exploit the higher apparent solubility and dissolution rate of the amorphous form. However, this may convert into the crystalline form over time as it is thermodynamically unstable [3,4]. A popular strategy to stabilise the amorphous form is to use amorphous solid dispersions in which the drug is molecularly dispersed in a water-soluble or water-swellable polymer. These are typically prepared by fusion (e.g., hot melt extrusion) or solvent evaporation (e.g., spray drying) methods [5,6].

In recent years, a number of studies have been conducted to understand the performance of amorphous solid dispersions during manufacturing, storage and *in vitro* dissolution [7,8,9,10]. The understanding of the formulation performance during dissolution is of particular interest as it underpins the *in vivo* efficacy. The dissolution mechanisms of amorphous solid dispersions are extremely difficult to deconvolute as a number of processes are involved [11]. Dissolution can be classified as drug-controlled or polymer-controlled depending on the physicochemical properties of the drug and the polymer, and the drug-to-polymer ratio [11]. The dissolution behaviour of amorphous solid dispersions can therefore be dependent on the polymeric carrier itself. The re-crystallisation of the drug in the solid state or from a super-saturated solution and the formation of drug nano- and micro-particles can also contribute to the final dissolution performance [9,10,12,13].

To gain a more complete picture of the drug release from amorphous solid dispersions, a number of solid-state analytical methods have been developed and employed, with a strong focus on how the solid state properties of the drug and polymer affect the drug release. These include ultraviolet (UV) imaging [14,15], infrared (IR) and Raman spectroscopy [10,12,16,17], magnetic resonance imaging (MRI) [9,18], ${}^{1}$H-NMR [19] and particle analysis (e.g., dynamic image analysis to monitor particle size and number during dissolution) [20]. UV imaging provides spatially and temporally resolved information, and in addition enables real time dissolution rates to be obtained. UV imaging has been employed to monitor in real time the drug re-crystallisation processes of several poorly soluble drugs [14,15]. IR and Raman spectroscopy can provide high chemical specificity (multi-variate data, one spectrum per xy position) [10,12,16,17]. Raman spectroscopy, compared to IR, is relatively insensitive to water which is clearly an advantage for imaging in aqueous environments. In addition, the low-wavenumber data of the Raman spectrum allows easy differentiation between amorphous and crystalline forms and between different polymorphic forms [10,21,22]. Coherent anti-Stokes Raman scattering (CARS), a particular form of Raman spectroscopy which uses two pulsed lasers focused on the sample to generate a signal, has also been employed to image the solid-state changes during dissolution of theophylline anhydrate [23]. In contrast, MRI offers relatively low chemical selectivity, but can provide three-dimensional information on molecular mobility. In recent work by Langham *et al*. magnetic resonance images were used to determine the erosion rates of a compact during dissolution [9].

Solution-state ${}^{1}$H-NMR has also recently been demonstrated to be a useful tool to monitor the dissolution of pharmaceutical products [19], especially for chemical species lacking a UV chromophore (e.g., soluble fillers), or medicines containing more than one active ingredient where quantification can be challenging using UV absorbance data. ${}^{1}$H-NMR offers a high chemical selectivity and therefore is capable of resolving signals from components through differences in their chemical shifts. In addition, as ${}^{1}$H-NMR spectra obtained from a dissolution test can be referenced against a spectrum of a solution with known concentration, quantification is possible [24,25].

While each of these techniques have been used to study the formulation dissolution performance, a better understanding of drug release can be achieved when techniques are used in combination.

**Figure 1:**
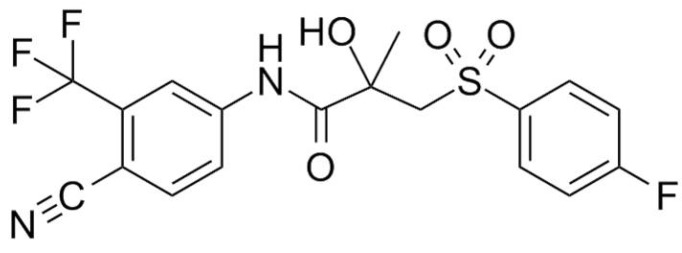
Molecular structure of bicalutamide.

We have previously investigated the dissolution performance of 5% and 50% drug-loaded bicalutamide-Kollidon VA64 extrudates using Raman mapping [12]. Bicalutamide (Figure 1) is an anti-androgen used for the treatment of prostate cancer and belongs to class II of the biopharmaceutics classification system (BCS), *i.e.*, it is characterised by low water solubility (less than 5 µg·$\;{\mathrm{mL}}^{-1}$) and high intestinal permeability [26,27]. Raman data indicated that amorphous bicalutamide present in the 50% extrudate re-crystallised into polymorphic forms II and I. As a result, the dissolution profiles of both bicalutamide and Kollidon VA64 (coPVP) were found to be extremely limited. We have also hypothesised that the re-crystallisation event follows the formation of an amorphous drug-rich shell due to the preferential dissolution of the hydrophilic polymer. In contrast, the dissolution performance of the 5% extrudate indicated the hydrophilic polymer and the hydrophobic drug dissolving at the same rate.

In the present paper we further explore the dissolution performance of bicalutamide: coPVP amorphous solid dispersions using a suite of analytical techniques. We investigated the 5% extrudate (low drug loading) and this time the 30% extrudate (intermediate drug loading), with the aim of obtaining additional information on how the drug amount affects the dissolution performance. A combined MRI/UV-Vis flow cell system was used, allowing changes in dissolution profile to be related to physical changes occuring in the solid material. Off-line ${}^{1}$H-NMR was also employed for the first time to simultaneously measure the dissolution profiles and rates of both the drug and the polymer from the molecular dispersion.

## 2. Results and Discussion

### 2.1. Solution-State Assays

#### 2.1.1. UV-Vis Flow Cell Experiments

**Figure 2:**
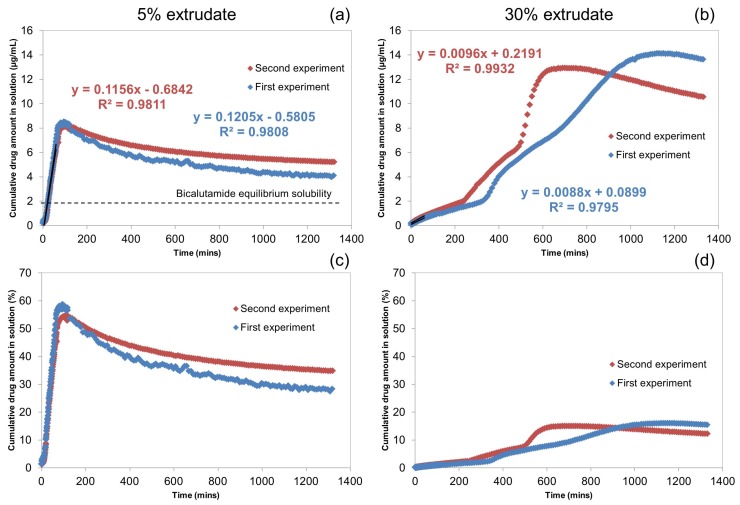
UV-Vis dissolution profiles of bicalutamide released from the 5% and 30% extrudates. (**a**,**c**) Drug release for the 5% extrudate expressed in Cumulative drug amount in solution (µg·$\;{\mathrm{mL}}^{-1}$) and Cumulative drug amount in solution (%) respectively; (**b**,**d**) Drug release for the 30% extrudate expressed in Cumulative drug amount in solution (µg·$\;{\mathrm{mL}}^{-1}$) and Cumulative drug amount in solution (%) respectively. The dissolution rates of the initial time-points were calculated using linear regression analysis. Linear best fits for the 5% and 30% dissolution experiments are included.

The UV-Vis dissolution profiles of the 5% and 30% extrudates are presented in Figure 2. The experiments were repeated twice for each drug loading to obtain an indication of the reproducibility in dissolution performance. A good reproducibility was observed in the two experiments of the 5% extrudate. Both profiles are characterised by two regions which correspond to the dissolving bicalutamide followed by the drug precipitation. In contrast, the two dissolution profiles of the 30% extrudate appear highly non-reproducible, *i.e.*, they are characterised by a different number of regions, but both show two pronounced “turning points” at 350 and 700 min (first experiment) and 250 and 500 min (second experiment) where the rate of drug dissolution increases. Overall, the UV-Vis data indicate a lack of controlled release for the 30% extrudate compared to the 5% extrudate.

These data indicate that there is a significant difference in dissolution behaviour between the two drug loadings. For example in the first experiment the 5% extrudate reaches a maximum concentration of 8.5 µg·$\;{\mathrm{mL}}^{-1}$ (which corresponds to approximately 59% of total release) at 95 min, with an initial dissolution rate of 0.12 µg·$\;{\mathrm{mL}}^{-1}$·$\;\min^{-1}$. The 30% extrudate achieves a maximum concentration in solution of 14.1 µg·$\;{\mathrm{mL}}^{-1}$ (≈16% of total release) at 1179 min and an initial dissolution rate of 0.01 µg·$\;{\mathrm{mL}}^{-1}$·$\;\min^{-1}$. For both drug loadings, the concentration of bicalutamide in solution declines after reaching the maximum concentration. The final measured concentration at 1319 min is 4.1 µg·$\;{\mathrm{mL}}^{-1}$ (≈28% of total release) and 13.6 µg·$\;{\mathrm{mL}}^{-1}$ (≈15% of total release) for the 5% extrudate and 30% extrudate respectively.

In accordance with previous work, the dissolution behaviour of the bicalutamide extrudates is clearly dependent on the drug loading [9,10,12]. At low drug loading (*i.e.*, 5% extrudate), the performance is dependent on the high aqueous solubility of the polymer. The drug is quickly released and the maximum concentration corresponding to approximately 59% of total release is reached within 95 min. For the extrudate containing a higher proportion of bicalutamide (*i.e.*, 30% extrudate), the dissolution performance is dominated by the physicochemical properties of the drug (e.g., low aqueous solubility and high hydrophobicity). As a result, the initial dissolution rate is approximately one order of magnitude lower (0.01 µg·$\;{\mathrm{mL}}^{-1}$·$\;\min^{-1}$ *vs.* 0.12 µg·$\;{\mathrm{mL}}^{-1}$·$\;\min^{-1}$) and the maximum concentration corresponding to 16% of total release is achieved only after 1179 min.

The UV-Vis data also show that the bicalutamide content in solution for both the 5% and 30% extrudates decreases after achieving the maximum concentration at 95 and 1179 min respectively, indicating that the drug super-saturation in solution is not maintained by the presence of coPVP across the entire dissolution test. In addition, in both experiments this reduction is accompanied by an increase of the absorbance at 350 nm (Figure S1 of SI), which is related to the scattering as well as absorption contributions of insoluble particles [13]. The formation of these particles, which cause the subsequent reduction in solution concentration, has been previously observed also for amorphous spray-dried solid dispersions of felodipine and coPVP [9].

It is also important to note that the 30% bicalutamide extrudate exhibited a markedly superior dissolution profile compared to the corresponding drug loading of amorphous spray-dried solid dispersions of felodipine and coPVP [9]. This can be attributed to the higher aqueous solubility of bicalutamide over felodipine [10,12], although the manufacturing route (hot melt extrusion *vs.* spray drying) may also be a factor.

#### 2.1.2. Off-Line ${}^{1}$H-NMR

**Figure 3:**
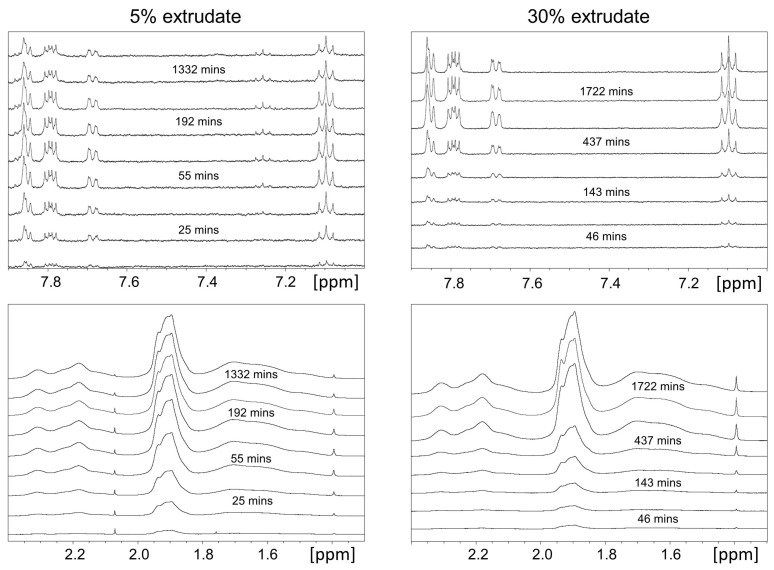
Portions of the ${}^{1}$H-NMR spectra acquired during the dissolution of the 5% and 30% extrudates. Bicalutamide and coPVP data were obtained from single integrals at 7.06–7.14 ppm (two aromatic protons of fluorobenzene) and 1.2–2.6 ppm (protons of pyrrolidone ring and methylene chains) respectively.

**Figure 4:**
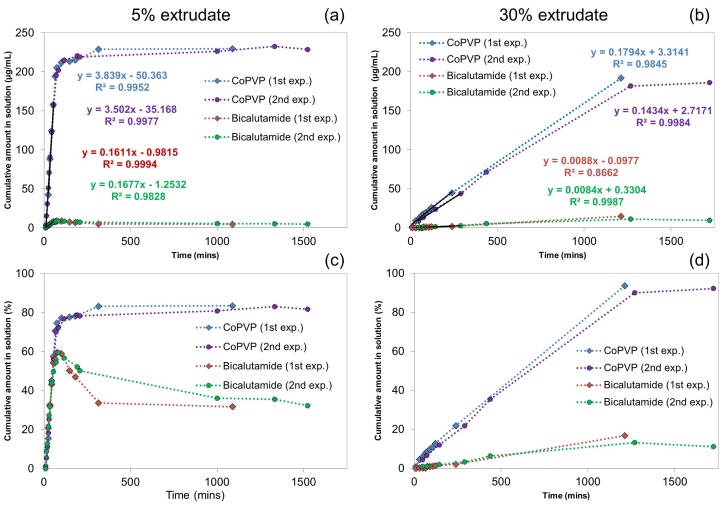
Release profiles obtained from the ${}^{1}$H-NMR spectra of bicalutamide and coPVP from the 5% and 30% extrudates. (**a**,**c**) Release for the 5% extrudate expressed in Cumulative drug amount in solution (µg·$\;{\mathrm{mL}}^{-1}$) and Cumulative drug amount in solution (%) respectively; (**b**,**d**) Release for the 30% extrudate expressed in Cumulative drug amount in solution (µg·$\;{\mathrm{mL}}^{-1}$) and Cumulative drug amount in solution (%) respectively. Data were scaled using the reference standard solutions. Linear best fits for the dissolution profiles of the 5% and 30% extrudates are included.

In this section we employ off-line ${}^{1}$H-NMR to complement the UV-Vis data for understanding the dissolution performance of the bicalutamide extrudates. With respect to standard UV-Vis measurements, ${}^{1}$H-NMR allows us to simultaneously determine the amount of drug and polymer in solution. As the dissolution performance of poorly soluble drugs can be largely dependent on that of the polymeric carrier, quantitative measurements of the latter provide valuable information [3,4]. In addition, similarly to the previously employed rotating disk dissolution rate (RDDR) methodology which uses HPLC to separate and therefore track the dissolution trends of both drug and polymer, the dissolution rate of the polymer allows us to determine an index of dissolution performance of the extrudate [10,12]. The index of performance shows how the drug and polymer behave during the dissolution test and it is defined by dividing the dissolution rate of bicalutamide by the total rate of the extrudate (bicalutamide plus coPVP) and normalising by the bicalutamide mass fraction (e.g., 0.05 for the 5% extrudate). The temporal evolution of NMR spectra for the 5% and 30% extrudates are presented in Figure 3, while the spectra from the reference solutions are shown in Figure S2 of SI. For each spectrum, two integrals from bicalutamide and coPVP were generated and then scaled to integrals obtained from the reference solutions of known concentration. The spectra from the reference solutions were obtained using identical experimental conditions. The release profiles of the individual species are shown in Figure 4.

For the 5% extrudate (e.g., first experiment), bicalutamide exhibits an initial dissolution rate of 0.16 µg·$\;{\mathrm{mL}}^{-1}$·$\;\min^{-1}$ and that of coPVP is 3.84 µg·$\;{\mathrm{mL}}^{-1}$·$\;\min^{-1}$. The index of performance resulted in a value of 0.8, indicating that the drug and polymer dissolve approximately with the same rate from the dispersion. The index of performance obtained from the ${}^{1}$H-NMR system is similar to that previously obtained from the RDDR test (value of 0.94) [12].

For the 30% extrudate, the dissolution rates of both bicalutamide and coPVP are lower with values of 0.009 µg·$\;{\mathrm{mL}}^{-1}$·$\;\min^{-1}$ and 0.18 µg·$\;{\mathrm{mL}}^{-1}$·$\;\min^{-1}$ respectively. In agreement with these data, the dissolution rates of bicalutamide and coPVP from the 50% extrudate were also found extremely low using the RDDR test [12]. The index of performance of the 30% extrudate is also far inferior (0.15) compared to that of the 5% extrudate, indicating that for this drug loading the higher content of bicalutamide limits the dissolution performance of both bicalutamide and coPVP.

The dissolution rates of bicalutamide obtained from the ${}^{1}$H-NMR experiments (0.16 $\;{\mathrm{mL}}^{-1}$·$\;\min^{-1}$ for the 5% extrudate and 0.009 $\;{\mathrm{mL}}^{-1}$·$\;\min^{-1}$ for the 30% extrudate) are in a good agreement with those obtained from the UV-Vis (0.12 $\;{\mathrm{mL}}^{-1}$·$\;\min^{-1}$ for the 5% extrudate and 0.009 $\;{\mathrm{mL}}^{-1}$·$\;\min^{-1}$ for the 30% extrudate).

From Figure 4 it is also apparent that for both the 5% and 30% extrudates, whilst coPVP achieves a dissolution release plateau, the bicalutamide concentration declines after reaching a maximum. In addition, for the 5% extrudate the bicalutamide precipitation begins before the coPVP dissolution is complete. This, in agreement with the previous UV-Vis data, clearly indicates that the super-saturation of the drug is not maintained by the polymer across the entire dissolution test.

### 2.2. Solid-State Assays

#### MRI Flow Cell Experiments

**Figure 5:**
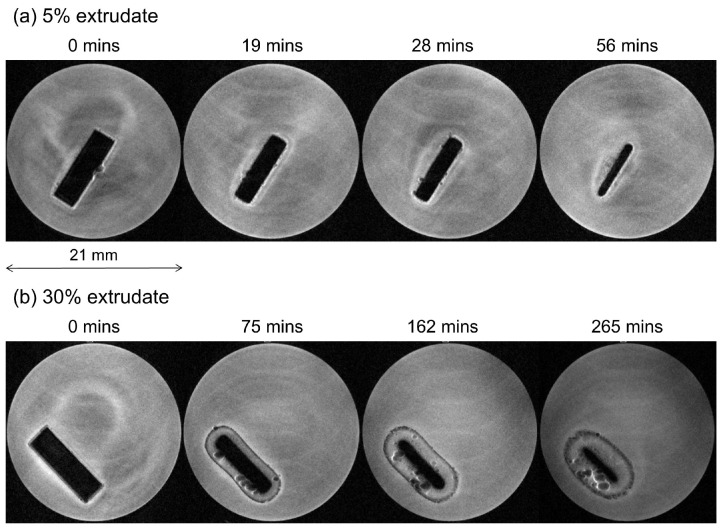
Magnetic resonance images (first experiment) showing the changes as a function of time in one cross sectional slice of the extrudates containing 5% (**a**) and 30% (**b**) of bicalutamide.

**Figure 6:**
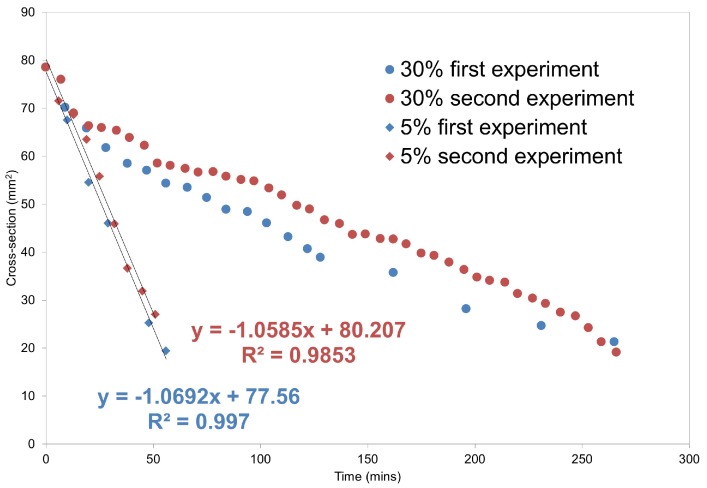
Evolution of the compact dimensions as a function of time for the 5% extrudate and 30% extrudate. Linear best fit to the data for the 5% extrudate is included. For the 30% extrudate, to facilitate the comparison with the 5% extrudate, the dimensions of the compact core (solid material) without considering those of the hydrated layer were measured. Data were collected only up to approximately 300 min due to the compact falling off from the support and being out of the instrumental field of view after this time-point.

The solution-state measurements obtained from the UV-Vis and ${}^{1}$H-NMR data are then complemented with solid-state measurements, *i.e.*, magnetic resonance imaging. The magnetic resonance images showing the temporal changes in one cross sectional slice of the 5% and 30% extrudates during the dissolution test are shown in Figure 5 (first experiment) and Figure S3 of SI (second experiment). The measured dimensions of the compacts as a function of time are presented in Figure 6. In this MRI experiment the image contrast is due to differences in molecular mobility. As a result, protons in a solid environment have a faster T${}_{2}$ relaxation time and therefore the solid materials appear dark in the images. Protons in solution have a significantly slower T${}_{2}$ relaxation time and thereby they generate a bright image, while, for example, protons in a hydrating gel give an intermediate brightness [9].

The images presented in Figure 5, for example, show significantly different dissolution behaviours for the 5% extrudate (Figure 5a) and 30% extrudate (Figure 5b). The 5% extrudate undergoes erosion and it is almost entirely dissolved over a period of 56 min, while the 30% extrudate swells and remains intact after 265 min (it is then no longer attached to the support due to its swelling and expansion and as a consequence it is out of the instrumental field of view). These results are in full accord with the UV-Vis and ${}^{1}$H-NMR solution-state data.

**Figure 7:**
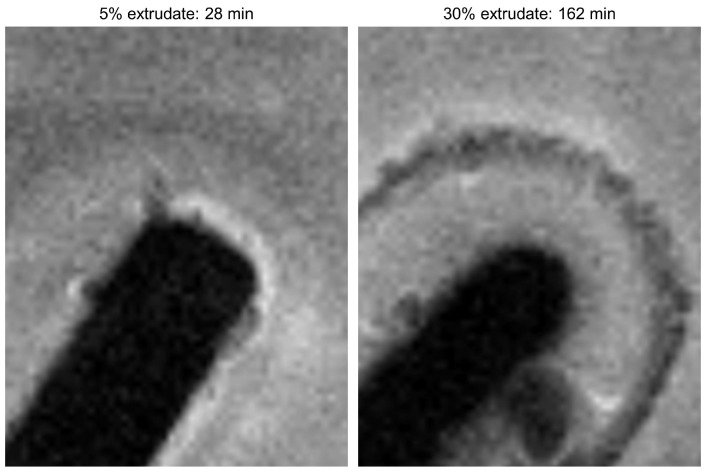
Zoom of the images collected for the 5% extrudate (28 min) and 30% extrudate (162 min) to highlight the well-defined region of intermediate MRI contrast present in the 30% extrudate.

Throughout the experiments, the 30% extrudate exhibits a well-defined region of intermediate MRI contrast, which is either not present, or hardly present, in the 5% extrudate (Figure 7). This region is due to water ingress into the compact. It is clear that for the 5% sample the rates of water ingress and dissolution are similar, which prevents the formation of the intermediate contrast region, whereas for the 30% compact dissolution is markedly slower than water ingress. Our previous Raman spectroscopy studies on a 50% sample [12] indicated that the preferential dissolution of coPVP led to formation of a drug-rich shell which crystallised and appeared to strongly inhibit further erosion and dissolution of the compact. We therefore suggest that this region of intermediate contrast in the magnetic resonance images of the 30% sample is due to water partially dissolved in the coPVP matrix, which does not lead to dissolution and drug release due to a drug-rich, likely crystalline shell which prevents further water ingress and thus greatly reduces the dissolution rate of the compact. Formation of a gel-layer can be ruled out on the basis of the optical images previously collected during the dissolution test of the 50% drug loading [12]. The drug re-crystallisation is likely to be one of the main factors which determines lack of controlled release observed in the UV-Vis profiles for the 30% drug loading.

It is important to note that the images in Figure 5 show some ghosting artefacts which are a likely consequence of the wash in/wash out effect of the flowing medium with nuclei moving between two consecutive spatially selective RF excitations. The artefacts do not interfere with the determination of the compact dimensions. The overall contrast of the flowing bulk dissolution medium seems to gradually decrease for the 30% loading sample and this is thought to be a consequence of the wash in/wash out effects of the flowing medium rather than due to any change in its mobility. If the intensity of the bulk medium in the initial image of 5% extrudate is compared with that for the 30% compact at 265 min they are similar. As the dissolution experiment proceeds, however, the difference in mobility and hence contrast between the hydrated compact and the bulk medium is reduced. In addition, bright halos on the outer edges of the compact as well as air bubbles are visible in the images of Figure 5. The origins of these are not fully understood but are likely to be due to magnetic susceptibility effects which are known to occur at interfaces. These effects are often observed with gradient echo imaging based sequences such as FLASH, which has been used in this work, and result in spatial mis-mapping of the MR signal, often presented as regions of very bright signal resulting from “piling up” of signal at the interface.

The difference in dissolution performance between the two drug loadings is reflected in Figure 6. The 5% extrudate quickly erodes with a linear erosion rate of 1.1 $\;{\mathrm{mm}}^{2}$·$\;\min^{-1}$, while the erosion rate for the 30% extrudate is slower and not linear due to the presence of the hydrating layer.

At the end of the dissolution test of the 30% extrudate (approximately after 1700 min), we noted abundant presence of undissolved material in the flow cell. This residue was removed from the flow cell and shown by Raman spectroscopy to contain crystalline bicalutamide form I (Figure S4), pointing to the drug re-crystallisation.

The MRI images are in agreement the UV-Vis and ${}^{1}$H-NMR data, indicating that for the 5% extrudate the release of bicalutamide is a polymer-controlled process whereby the dissolution is governed by the fast hydration of the highly water-soluble coPVP. For the 30% extrudate, the dissolution is dependent on the physicochemical properties of bicalutamide. The low aqueous solubility and high hydrophobicity of the drug contribute to a slower water uptake into the compact which corresponds to a slower dissolution rate of both bicalutamide and coPVP.

## 3. Experimental Section

### 3.1. Materials

Bicalutamide was provided by AstraZeneca (Macclesfield, UK) and shown to be polymorphic form I by Raman spectroscopy and X-ray powder diffraction (XRPD). Kollidon VA64 (coPVP) was supplied by BASF (Ludwigshafen, Germany). All materials were used as received and without any further purification.

### 3.2. Sample Preparation

#### 3.2.1. Preparation of the Amorphous Reference form of Bicalutamide

The amorphous form of bicalutamide was prepared by heating form I to 200 C and quench cooling the melt to room temperature [12]. The formation of the amorphous form was confirmed by Raman spectroscopy and XRPD (Figures S4 and S5 of SI).

#### 3.2.2. Preparation of the Bicalutamide: coPVP Extrudates

Extrudates of bicalutamide in coPVP (5% and 30% *w*/*w* bicalutamide) were prepared using a co-rotating twin-screw extruder (Thermo Scientific HAAKE MiniLab II). Bicalutamide form I and coPVP were pre-mixed for 20 min in a Turbula T2F mixer (Willy A. Bachofen AG Mashinefabrik). The materials were extruded at a screw speed of 150 rpm and a temperature of 170 °C, then cooled to room temperature and manually milled with a T & G CrushGrind mill to fine powders. The formation of the amorphous form was confirmed by Raman spectroscopy and XRPD (Figures S4 and S5 of SI).

### 3.3. Analytical Methods

#### 3.3.1. Raman Spectroscopy

Data were acquired on a HORIBA Jobin Yvon LabRAM HR confocal microscope/spectrometer. A near-IR (785 nm) laser was employed to illuminate the samples, and spectra were collected using a 50× objective and a 300 µm confocal pinhole. A 600 lines·mm${}^{-1}$ rotatable diffraction grating along a path length of 800 mm was used to simultaneously scan a range of Raman shifts, and spectra were detected using a SYNAPSE CCD detector (1024 pixels). Spectra were collected in the range from 1055 to 1725 $\;{\mathrm{cm}}^{-1}$.

#### 3.3.2. X-ray Powder Diffraction (XRPD)

X-ray powder diffraction data were collected on a PANalytical CubiX PRO diffractometer (λ = 1.5418 Å). Powders were smeared onto zero-background silicon wafer sample holders and measured over the scan range from 2${}^{\circ }$ to 40${}^{\circ }$ 2θ, with a 25 s exposure per 0.02${}^{\circ }$ 2θ increment. Samples were spun at 30 rpm and exposed to a radiation generated by a copper long-fine focus tube operated at a voltage of 45 kV and a current of 40 mA.

#### 3.3.3. MRI UV-Vis Flow Cell System

The dissolution performance of circular compacts with a diameter of 10 mm and a weight of approximately 290 mg was investigated. Powders were compressed using a Specac manual hydraulic press using a compression force of *ca.* 50 kN. The dissolution tests were performed in a USP IV-type flow cell previously described by Langham *et al*. [9]. 1 L of pH 6.5 phosphate buffer dissolution medium maintained at a temperature of 37 °C using a temperature-controlled water bath was circulated continuously at a flow rate of 15 mL·min${}^{-1}$.

The dissolution profile of bicalutamide was obtained by recording the absorbance of the unfiltered dissolution medium at 275 nm (wavelength maxima of bicalutamide) and 350 nm (no observed absorbance, for correction for any suspended particles) at regular time intervals using a Agilent 8453 UV-Vis spectrophotometer with a 10 mm quartz flow cell (Starna Scientific, Ilford, UK). The dissolution data were calibrated against the absorbance of a series of standard solutions of bicalutamide in water–acetonitrile 75/25 (*v*/*v*).

The flow cell was sited in the probe of a 400 MHz Bruker Avance NMR spectrometer fitted with a Micro2.5 imaging accessory fitted with a 25 mm O.D. RF coil. The images were collected as a function of time using a FLASH (Fast Low Angle Shot) protocol to generate 16 × 1 mm${}^{2}$ concatenated axial slices with a field of view of 25 mm. The echo time was 3.0 ms and the repetition time was 500 ms giving a scan time of 1 min 4 s for each 16 slice experiment. A data matrix size of 128 × 128 pixels was recorded giving a resolution of 0.0195 cm/pixel in the read direction. Data were processed using ParaVision™ software v4.0. Changes in the size of the compacts were followed by measuring the dimensions of a single slice as a function of time.

#### 3.3.4. ${}^{1}$H-NMR Spectroscopy

All NMR measurements were performed on a Bruker Avance III 500 MHz NMR spectrometer fitted with a 5 mm QCI cryoprobe. The spectra from the dissolution samples and the reference solutions were acquired at 300.0 K with a spectral width of 10 kHz, with 64 k data points in the time domain. A recycle delay of 4 s was used and the peak due to water was suppressed using a 1D NOESY pulse program with presaturation and spoil gradients (noesygppr1d) and with irradiation at the water frequency during the recycle and mixing time delays. The receiver gain was set to 128 and kept the same for each experiment. Data were processed using the Bruker Topspin 3.0 software. Interactive zero order phase correction was applied to all the spectra and the “Use lastscale for calibration” functionality was employed to directly compare integrals across multiple spectra.

## 4. Conclusions

A combination of solution-state and solid-state analytical techniques were employed to fully understand the dissolution performance of amorphous bicalutamide solid dispersions at two different drug loadings (5% and 30% bicalutamide). The integrated MRI UV-Vis system enabled us to relate changes in dissolution profile to physical changes occurring to the solid material. The MRI data indicated that the 5% extrudate erodes linearly, while for the 30% extrudate the water ingress is significantly slower which corresponds to a slower dissolution of both bicalutamide and coPVP. We also employed for the first time ${}^{1}$H-NMR spectroscopy to simultaneously measure the dissolution profiles and rates of both drug and polymer. ${}^{1}$H-NMR has been demonstrated to be a valid alternative to the previously employed rotating disk dissolution rate (RDDR) methodology for tracking the dissolution profiles in multi-component systems. ${}^{1}$H-NMR data showed that for the 5% extrudate, bicalutamide and coPVP dissolve with approximately the same rate pointing to a matrix-controlled release, while for the 30% drug loading they dissolve very differently and at a significantly lower rate. For the 30% extrudate the dissolution performance is dominated by the physicochemical properties of the drug.

## Supplementary Materials

Supplementary materials can be accessed at: http://www.mdpi.com/1420-3049/20/09/16404/s1.
