# Supplementary material for: Investigating the Dissolution Performance of Amorphous Solid Dispersions Using Magnetic Resonance Imaging and Proton NMR
Source: Molecules. 2015 Sep 10;20(9):16404–18. doi: 10.3390/molecules200916404 (PMC6331940; doi:10.3390/molecules200916404)
Supplement: Supplementary file 1 [file molecules-20-16404-s001.pdf]

Supplementary Information

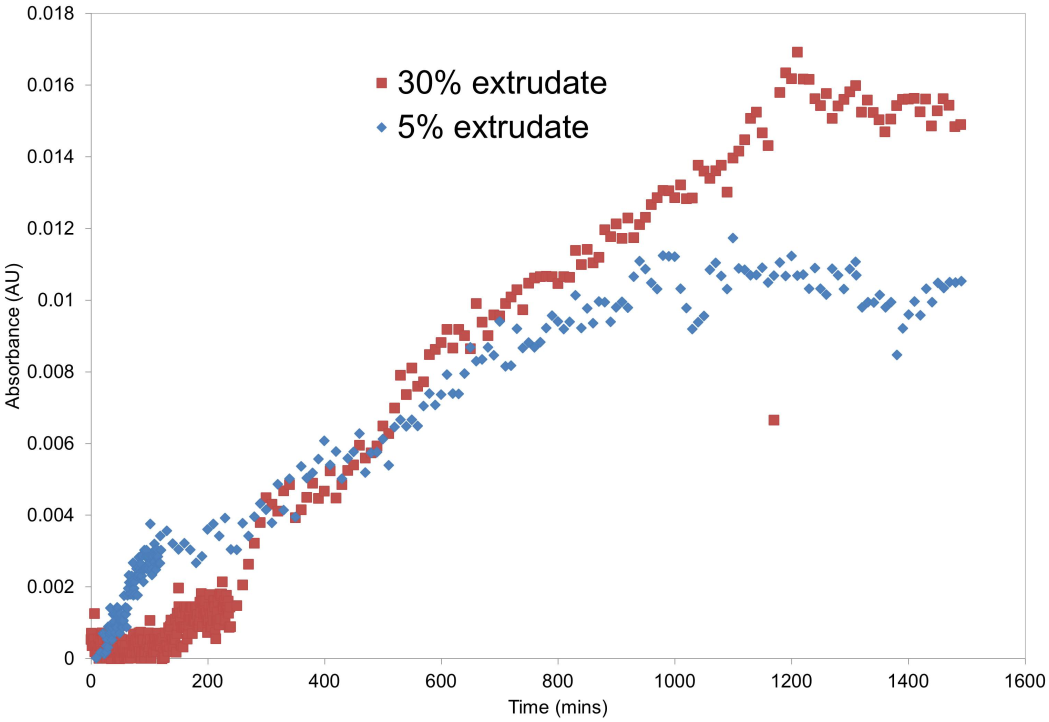

Figure S1. Absorbance UV-Vis values at 350 nm acquired during the dissolution of the 5% and 30% extrudates.

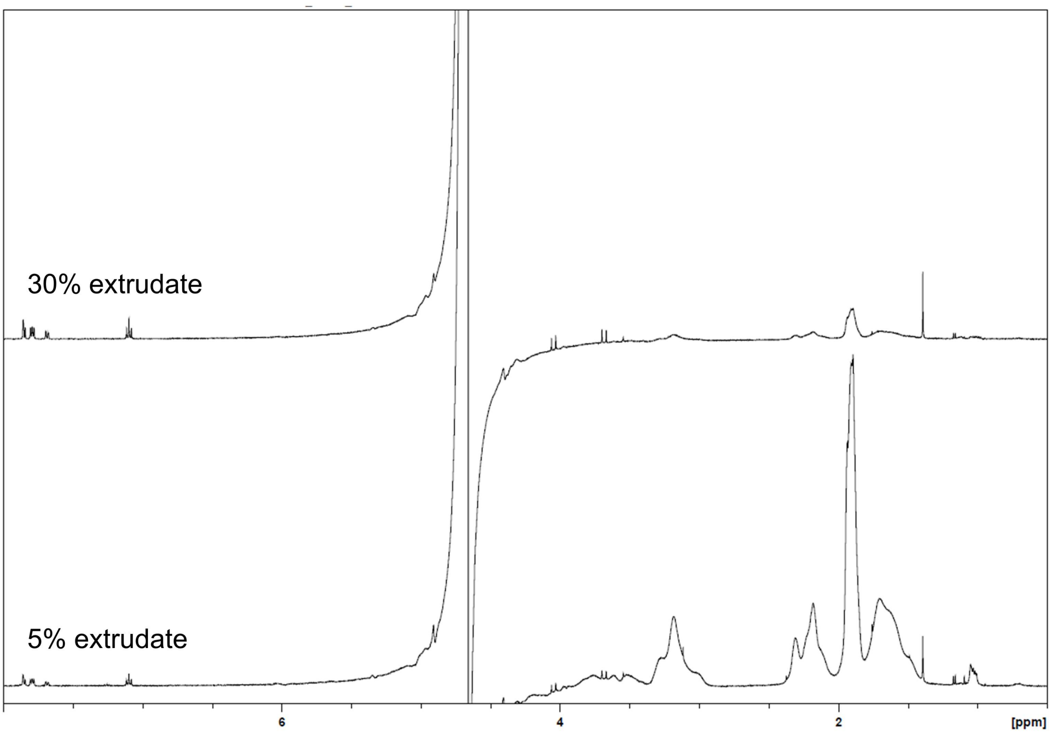

Figure S2. ^{1}H-NMR spectra of reference solutions of 5% and 30% extrudates.

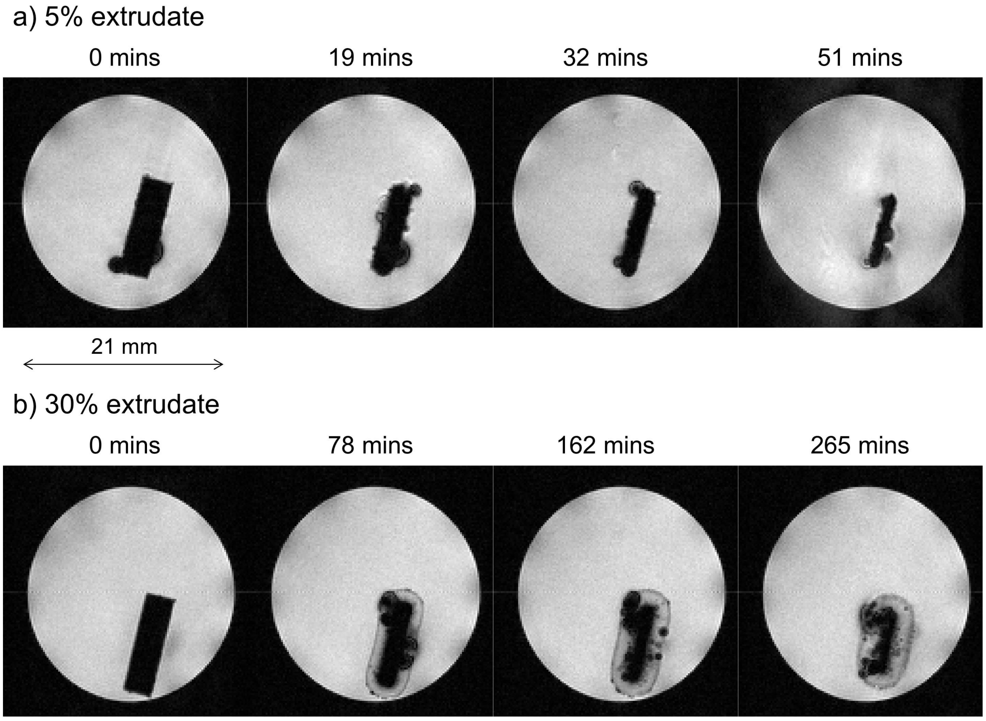

Figure S3. Magnetic resonance images (second experiment) showing the changes as a function of time in one cross sectional slice of the extrudates containing 5% (**a**) and 30% (**b**) of bicalutamide.

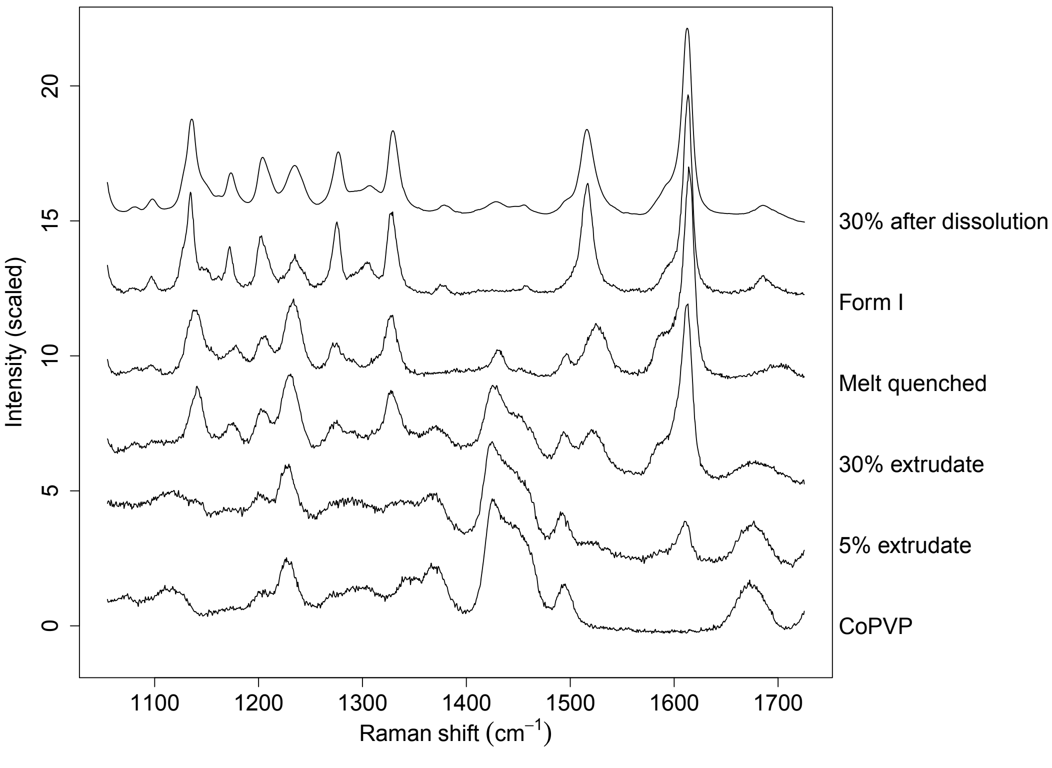

Figure S4. Variance-scaled and mean-centred Raman spectra of all raw materials and the 30% extrudate residue recovered after the dissolution experiment.

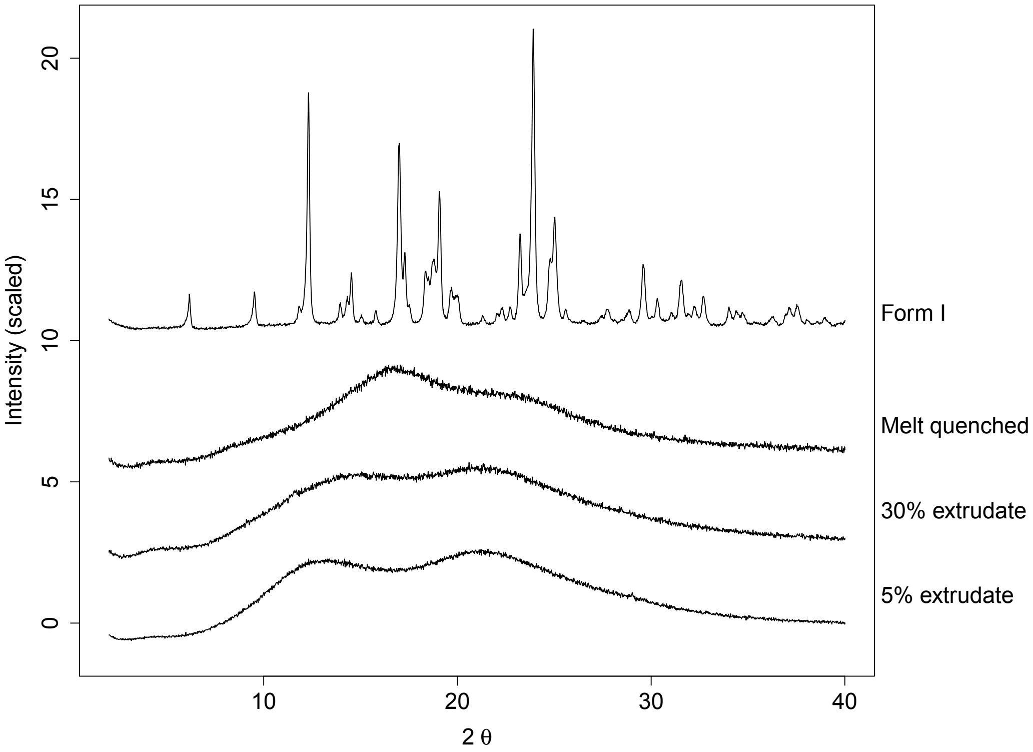

Figure S5. Variance-scaled and mean-centred XRPD patterns of all raw materials.

© 2015 by the authors; licensee MDPI, Basel, Switzerland. This article is an open access article distributed under the terms and conditions of the Creative Commons Attribution license (http://creativecommons.org/licenses/by/4.0/).
